# MiRNA-141 and miRNA-200b are closely related to invasive ability and considered as decision-making biomarkers for the extent of PLND during cystectomy

**DOI:** 10.1186/s12885-015-1110-7

**Published:** 2015-03-04

**Authors:** Wentao Liu, Lin Qi, Hui Lv, Xiongbing Zu, Minfeng Chen, Jun Wang, Longfei Liu, Feng Zeng, Yuan Li

**Affiliations:** 1 Department of Urology, Xiangya Hospital, Central South University, No.87 Xiangya Road, Changsha City, Hunan Province 410008 P. R. China; 2 Department of Pathology, Xiangya School of Medicine, Central South University, Changsha City, Hunan Province P. R. China; 3 Department of Urology, First Teaching Hospital, Zhengzhou University, Zhengzhou City, Henan Province P. R. China; 4 Present address: Department of Urology, The second Xiangya Hospital, Central South University, Changsha City, Hunan Province P. R. China

**Keywords:** microRNA, Invasive, EMT, Bladder cancer, Lymph node dissection

## Abstract

**Background:**

MicroRNAs (miRNAs) are small non-coding RNAs that silence their cognate target genes by specifically binding and cleaving messenger RNAs or inhibiting their translation. In this study, we explored whether miRNA-141 and miRNA-200b are involved in regulation of the invasive ability and epithelial–mesenchymal transition (EMT) of bladder cancer cells in vitro. We also evaluated their potential as biomarkers for deciding the extent of pelvic lymph node dissection (PLND) required during radical cystectomy.

**Methods:**

Pri- and anti-miR cell lines were constructed. The invasive capacity of the cells was tested using a cell invasion assay. The MMP-2, MMP-9 and EMT-related markers were validated through Western blotting analysis. Seventy-eight urine samples from patients undergoing cystectomy and super-extended lymph node dissection were evaluated by qRT-PCR.

**Results:**

Loss of expression of miRNA-141 and miRNA-200b was associated with increased invasion and migration ability, upregulated MMP-2, MMP-9, vimentin and N-cadherin expression, and downregulated E-cadherin expression in bladder cancer cell lines. Urine miRNA-141 and miRNA-200b levels could discriminate patients with lymph node metastasis from those who were lymph node negative (AUC: 0.704 and 0.674, respectively).

**Conclusion:**

MiRNA-141 and miRNA-200b play important roles in the invasive ability and EMT phenotype of bladder cancer. Detection of miRNA-141 and miRNA-200b can help to identify patients undergoing cystectomy who are likely to have lymph node metastasis, and therefore those who may benefit from super-extended PLND.

**Electronic supplementary material:**

The online version of this article (doi:10.1186/s12885-015-1110-7) contains supplementary material, which is available to authorized users.

## Background

Bladder cancer (BC) is the 6th most common cancer in the US, with 72,570 cases estimated in 2013 [1]. Patients diagnosed with superficial bladder cancer do not face a life-threatening situation; however, up to 70% of these patients will develop at least one recurrence within 5 years [2]. Patients with locally advanced or metastatic bladder cancer show disappointingly low 5-year overall survival (OS) rates, at 10–15% [3]. Currently, radical cystectomy plus pelvic lymph node dissection (PLND) is considered to be a standard treatment for muscle-invasive bladder cancer and some high-risk non-muscle-invasive bladder cancers [4].

The use of PLND may provide not only therapeutic but also diagnostic benefits [5]. The risk of lymph node metastasis (LN+) in muscle-invasive bladder cancer is up to 24% [6], and it is mainly associated with tumor invasion [7]. Currently, reports on the extent of PLND have not reached a consensus. Some urologists have used imaging of the primary tumor or enlarged lymph nodes on computer tomography (CT) to predict LN+, but this technique is limited owing to its relatively low diagnostic accuracy [4]. Recently, numerous studies have recommend super-extended PLND (to the origin of the inferior mesenteric artery from the aorta) for all patients with BC who are undergoing radical cystectomy [8,9]. Given the longer surgical procedure and greater blood loss associated with super-extended PLND, it remains uncertain whether all patients need it.

MicroRNAs (miRNA) are small non-coding RNAs that silence their cognate target genes by specifically binding and cleaving messenger RNAs, or inhibiting their translation [10]. MiRNAs are involved in various biological processes, including cell cycle control, apoptosis, cell proliferation and invasion [11]. Recently, some miRNAs detected in urine have been considered as urinary biomarkers for BC [12]. The miRNA-200 family is composed of five members, arranged as two clusters: miRNA-200a/200b/429 and miRNA-200c/141 [13]. The miRNA-200 family is considered to be involved in the early stages of tumor metastasis [14]. Loss of expression of the miRNA-200 family (miRNA-200a, −200b, −200c, −141 and- 429) has been reported in several types of advanced carcinoma, including BC [15-17].

In this study, we first identified that miRNA-141 and miRNA-200b are the most differentially expressed miRNAs among the miRNA-200 family members. We explored whether they were involved in the regulation of invasive ability and epithelial–mesenchymal transition (EMT) of BC cell in vitro. We also evaluated their potential as biomarkers for deciding the extent of PLND required during cystectomy.

## Methods

### Fresh tissue

This study was approved by the Ethics Committee of Central South University, Changsha city, Hunan province, China. Informed consent was obtained from all of the patients. The methods were carried out in accordance with the approved guidelines. Specimens of BC and corresponding adjacent tissues were collected from 30 patients with muscle-invasive BC who underwent cystectomy at Xiangya hospital, Central South University.

### MiRNA expression analysis

To investigate the differential expression of five members of the miRNA-200 family in BC tissues and adjacent tissues, we analyzed 60 fresh bladder tissues (30 BC specimens and 30 corresponding adjacent tissues) using qRT-PCR. For RNA extraction, bladder tissues with a diameter of about 3–4 mm were used for RNA extraction with a mirVana™ miRNA Isolation Kit, following the instructions of the manufacturer (Applied Biosystems, USA). For detection of miRNAs, qRT-PCR was performed using TaqMan miRNA assays (Applied Biosystems, USA) with specific commercial primer sets and probes. All reagents and protocols were obtained from Applied Biosystems and detection was performed using U6 as an internal control. Comparative quantification was performed on the basis of a 2^−ΔΔCt^ calculation method [18]. The miRNA specific qRT-PCR was done in triplicate and repeated three times.

### Cell culture

The human bladder carcinoma cell lines CRL 1749, J82, T24, HT 1376 and HTB 9 were maintained in Dulbecco’s Modified Eagle’s Medium (DMEM) supplemented with 10% fetal bovine serum and penicillin/streptomycin.

### Establishment of stable cell lines

To explore the function of endogenous miRNA-141 and miRNA-200b, we generated constructs of pri- or anti-miRNA, aiming either to increase or to inhibit the function of the molecules. A miRNA sponge was initially developed by Ebert and colleagues to inhibit miRNA function [8]. The pLVX-IRES-ZsGreen1 expression vector was purchased from Clontech Laboratories, Inc (USA).

Lentiviral vectors of pLVX-pri-miRNA-141 or pLVX-pri-miRNA-200b which expressed miRNA-141 or miRNA-200b were constructed (Yinrunbio, Changsha, China). Sponge plasmids, pLVX-miRNA141-sponge and pLVX-miRNA200b-sponge, were constructed as described by Ebert et al. [8].

A pre-mixed Lentiviral Packaging System (Biosettia, SD, USA) was used for viral packaging. Briefly, lentivirus was produced by transfection of 293 T cells at 5 × 10^6^ cells/10 cm plate using Lipofectamine 2000. Supernatants were collected 48 h after transfection and filtered; the viral titers were determined by fluorescence-activated cell sorting (FACS) at 48 h post-transduction. The BC cells were infected with lentivirus in the presence of 8 μg/ml polybrene (Sigma-Aldrich, USA). The green fluorescence protein (ZsGreen1), which was co-expressed in lentivirus-infected cells, served as a selection marker to indicate the successfully infected cancer cells.

### Cell invasion assay

The invasive capacity of the cells was determined using BD BioCoat Matrigel invasion chambers (8-μm pores) (BD Biosciences, USA). The cells were seeded on the top chamber, incubated at 37°C, and allowed to invade and migrate through the Matrigel and the membrane pores in the inserts. After 48 h, the cells on the surface of the membrane were wiped off. The cells on the underside of the membrane were fixed and stained. The invasive cells were counted under a microscope at 100× magnification.

### qRT-PCR for mRNA study

Total RNA from cell samples was extracted using an RNeasy Mini Kit (Qiagen, USA). Complementary DNA (cDNA) was synthesized using the High-Capacity cDNA archive kit (Applied Biosystems, USA), according to the manufacturer’s protocol. The cDNA was synthesized from 2 μg total RNA on a PTC-200 Peltier Thermal Cycler DNA Engine (MJ Research Inc., USA). The DNA Engine Thermal Cycler with Chromo 4™ real-time detector system and Opticon Monitor software (Bio-Rad Laboratories, USA) were used for real-time PCR analysis. Cycle threshold (Ct) values were normalized to the housekeeper GAPDH gene.

The specific primers used were as follows: GAPDH (forward: 5′-ACCACAGTCCATGCCAT CAC-3′; reverse: 5′-TCCACCACCCTGTTGCTGTA-3′); N-cadherin (forward: 5′-AACCCTTATTTT GCCCCCAAT-3′; reverse: 5′-TCAACATGGTACCGGCATGA-3′); E-cadherin (forward: 5′-CGGGA ATGCAGTTGAGGATC-3′; reverse: 5′-AGGATGGTGTAAGCGATGGC-3′); vimentin (forward: 5′-GACCTCTACGAGGAGGAGAT-3′; reverse: 5′-TTGTCAACATCCTGTCTGAA-3′).

### Western blotting analysis

Cultured cells were directly lysed for 30 minutes on ice with lysis buffer [50 mmol/L Tris–HCl (pH 7.4), 1% Nonidet P-40, 0.25% sodium deoxycholate, 150 mmol/L NaCl, 1 mmol/L EDTA, 1 mmol/L PMSF, 1 μg/mL aprotinin, 1 μg/mL leupeptin, 1 μg/mL pepstatin, 1 mmol/L Na_3_VO_4_ and 1 mmol/L NaF]. After centrifugation at 13,000 *g* for 15 min, protein concentrations were measured using Bradford’s reagent (Bio-Rad laboratories, USA), and the protein was denatured by boiling for 10 min. Protein (25 μg) was loaded onto sodium dodecylsulfate–polyacrylamide gels for electrophoresis and then transferred onto nitrocellulose membranes. After blocking with 5% milk in TBST (137 mmol/L NaCl, 25 mmol/L Tris, and 1 mmol/L disodium ethylenediaminotetraacetate containing 0.1% Tween-20), the membranes were incubated with anti-E-cadherin, anti-vimentin (Cell Signaling Technology, USA), anti-matrix metalloproteinase (MMP)-2 and MMP-9 (Chemicon, USA), anti-N-cadherin (Santa Cruz Biotechnology, USA), and anti-GAPDH (Santa Cruz Biotechnology, USA) at 4°C overnight. After washing with TBST three times (10 min each), the membranes were incubated with their corresponding horseradish peroxidase (HRP)-conjugated secondary antibodies at room temperature for 1 h. After washing with TBST three times (10 min each), bound antibodies were visualized using enhanced chemiluminescent substrates (Amersham Bioscience, USA).

### Gelatine zymography

Gelatine zymography was used to evaluate the levels of expression of MMPs in conditioned media from cultured cells. To generate supernatants for zymography, cells were seeded in six-well plates with complete medium. On the following day, the medium was replaced by starving medium containing 0.1% BSA. After incubation for 24 h, supernatants were collected, centrifuged and analyzed by zymography. The conditioned medium was electrophoresed in a polyacrylamide gel containing gelatin at a concentration of 1 mg/mL. The gel was washed at room temperature for 2 h with 2.5% Triton X-100 and then at 37°C overnight in a buffer containing 10 mmol CaCl2, 150 mm NaCl and 50 mmol Tris–HCl (pH 7.5). For visualization of gelatinolytic activity, the gels were stained with Coomassie blue and photographed on a light box. Proteolysis was detected as a white zone in a dark blue field.

### Patients and urine samples

Urine samples from 78 patients with BC were obtained from our center between January 2010 and March 2013. Agreement of collecting their urine and written informed consent have been obtained from all included patients. All patients underwent laparoscopic cystectomy accompanied by super-extended PLND as described in a previous report [19]. Their average age was 57 + 12.7 years. Sixty-four (82.1%) of the patients were male and 14 (17.9%) were female. Radiological tests, including chest X-ray and CT**,** were routinely done according to EAU guidelines [20]. Preoperative CT staging of bladder tumors was evaluated using a standardized method [19]. Histopathological examination of bladder cancer specimens and lymphatic tissues obtained during lymph node dissection was performed by experienced genitourinary pathologists. The region of lymph node metastasis (LN+) was divided into level I, level II and level III, as introduced by Leissner et al. [8].

Total urine samples (100–150 mL) were collected before cystectomy. The urine was stored at 4°C for up to 4 h and then centrifuged. The pellet was re-suspended in 1 ml of TRIzol reagent and frozen at −80 C in liquid nitrogen for further use. RNAs were extracted from the urinary cell pellets using a mirVana™ miRNA Isolation Kit, following the instructions of the manufacturer (Applied Biosystems, USA). The expression of miRNA-200b and miRNA-141 was detected using qRT-PCR as described above.

### Statistical analysis

A two-tailed chi-square test was used to determine the statistical significance of differences between proportions. The Mann–Whitney U test or Wilcoxon signed-rank test was used for continuous variables. The value of each biomarker in deciding the extent of PLND was evaluated by calculating the ROC AUC. The ROC AUC of each model was compared using the DeLong test [21]. P values less than 0.05 were counted as significant in all tests. The statistical analysis was performed using SPSS for Windows v.13.0 and MedCalc statistical software 11.5.0.

## Results

### Quantification of the miRNA-200 family in bladder cancer tissues

To identify the specific miRNA-200 family member relevant to invasiveness and metastasis of BC, we used qTR-PCR to measure the expression of five members of the miRNA-200 family in muscle-invasive BC specimens and corresponding adjacent tissues. Additional file 1: Table S1 lists details of the expression of the miRNA-200 family. Compared with adjacent tissues, expression of the miRNA-200 family was deregulated in specimens from muscle-invasive bladder carcinoma (1.41- to 4.28-fold change). The most significantly regulated miRNAs in our series were miRNA-141 (3.25-fold) and miRNA-200b (4.28-fold). Therefore, we selected these two miRNAs for further in vitro investigation to confirm their effect on the invasive ability of malignant bladder cells and to explore whether they are involved in the regulation of EMT.

### Quantification of the miRNA-200 family in a panel of five bladder cancer cell lines and stable lentiviral transduction of pri- or anti-miRNAs targeting miRNA-141 or miRNA-200b in CRL1749 and HTB9 cells

**Figure 1:**
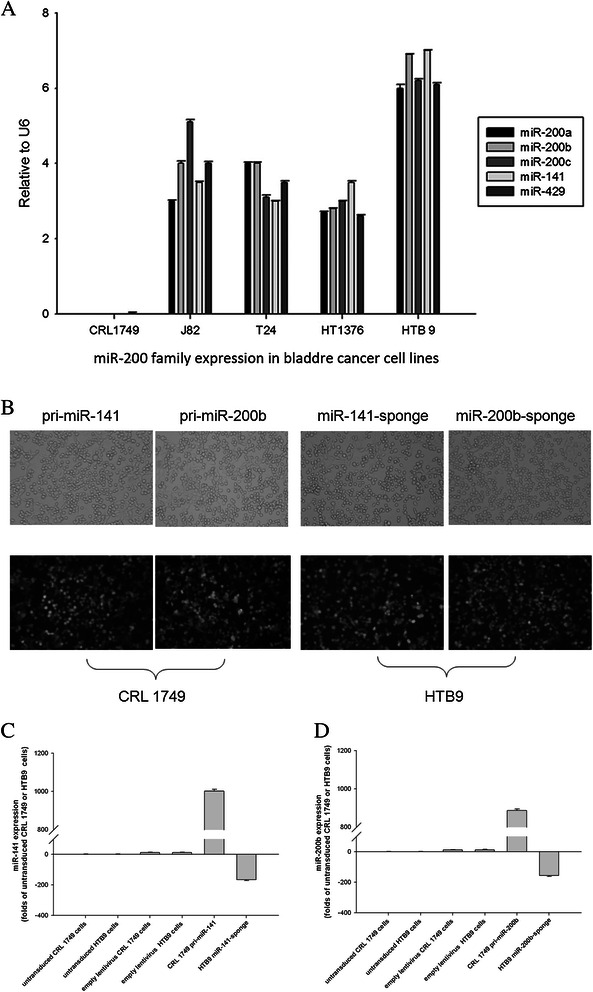
**Expression of miRNA 200 family in bladder cell lines and in vitro lentiviral transduction in bladder cell lines.** Expression (±SD) of miRNA-200a, −200b, −200c, −141 and 429 in a panel of 5 bladder cancer cell lines was determined by TaqMan miRNA qRT-PCR assays **(A)**. Mean of triplicate RT-PCR assays. Expression was normalized to U6 (2^−ΔCt^). Stable lentiviral transduction of pri-miRNA-141 and -200b into CRL 1749 cells, and miRNA-141-sponge and miRNA-200b-sponge into HTB9 cells was performed. Transduction efficiency was determined by fluorescent microscopy after transduction with lentivector encoding green fluorescence protein. Over 80% of transduced cells showed green fluorescent signals. Magnification was 200× **(B)**. The expression levels of miR-141 or -200b were determined by RT-PCR **(C and D)**. The expression of miR-141/200b was significantly overexpressed in CRL 1749 pri-miR-141/200b cells, while greatly repressed in HTB9 miR-141/200b-sponge cells when compared with respective untransduced and empty lentivirus control cells (**P < 0.01). CRL 1749 pri-miR-141/200b or HTB9 miR-141/200b-sponge cells referred to cells stably transduced with lentivirus encoding pri-miR-141/200b or miR-141/200b -sponge.

We observed various levels of expression of the miRNA-200 family in these cell lines (Figure 1A). Five members of the miRNA-200 family were almost undetectable in CRL 1749 cells. The highest expression of the five members was observed in cells of the HTB9 line. Based on these findings, the CRL 1749 and HTB9 cell lines were selected for further study. MiRNA-141 and miRNA-200b were observed to have the highest expression among the five miRNAs in the HTB9 cells. MiRNA-141 and miRNA-200b were almost undetectable in CRL 1749 cells.

The lentiviral transduction efficiency of CRL1749 and HTB9 cells was determined by the detection of ZsGreen1 signals using fluorescence microscopy at 72 h after transduction, and was confirmed to be >80% (Figure 1B). FACS was performed to select stably transduced cells. After cell sorting, the expression of miR-141 or miR-200b in stably transduced cells was measured by real-time PCR. The expression of miR-141 and miR-200b in CRL 1749 pri-miR-141/200b cells was upregulated 100.2- and 88.6-fold, respectively, when compared with untransduced control cells (P < 0.01); this was confirmed by RT-PCR in addition to the FACS observation (Figure 1C and D). In contrast, the expression of both miRNAs was greatly repressed in HTB9 141/200b-sponge cells. The repression was 16.6- and 15.5-fold, respectively, when compared with untransduced HTB9 cells (P < 0.01) (Figure 1C and D).

### Manipulation of the expression of miRNA-141 or miRNA-200b can change the invasive potential and expression of MMP-2 and −9

**Figure 2:**
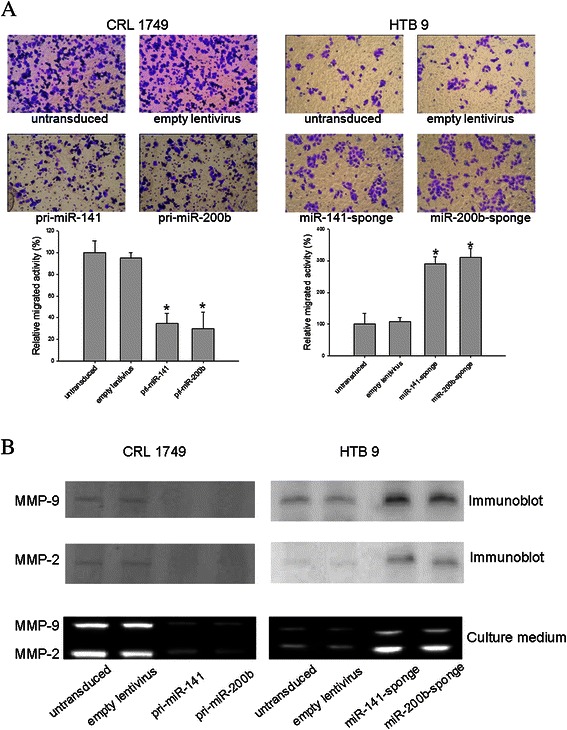
**Modulation of expression of miRNA-141 or miRNA-200b in CRL 2749 and HTB9 leaded to changes of MMPs expression and the invasive potential.** Transduced and untransduced cells were plated on the top chamber for 48 h, before photos were taken of invaded cells. Results were expressed as the number of invaded cells relative to untransduced control cells, as determined from three independent experiments **(A)**. *P < 0.05 compared with untransduced control. Gelatine zymography was performed with cell supernatants harvested after 24 h of cultivation. Gelatinolytic activity was attributed to the activity of MMP-2 and MMP-9 **(B)**. Cell lysates of untransduced and transduced cells were subjected to immunoblot analysis for MMP-2, MMP-9 and GAPDH **(B)**.

To explore whether modulation of miRNA-141 and miRNA-200b levels affects invasive potential, we performed the matrigel invasion chamber assay. As shown in Figure 2A, the invasion and migration of CRL 1749 cells was inhibited by forced overexpression of miRNA-141 or miRNA-200b. Repression of miRNA-141 or miRNA-200b in HTB9 cells strongly increased invasion and migration (Figure 2A). Gelatine zymography detected changes in MMP-2 and −9 expression in cell supernatants from two human BC cell lines (CRL 1749 and HTB9 cells) that were either transduced or not (Figure 2B). The gelatinolytic activity of the MMP-2 and MMP-9 enzymes was reduced markedly when CRL 1749 cells were transduced with pri-miRNA-141 or pri-miRNA-200b-encoding lentiviral particles. In contrast, the activity of the enzymes was upregulated in HTB9 cells transduced with lentivirus expressing miRNA-141-sponge or miRNA-200b-sponge. Similar results were found for the lysates and immunoblotting results (Figure 2B).

### Activities of the EMT-related molecules E-cadherin, vimentin and N-cadherin were changed at mRNA and protein levels

**Figure 3:**
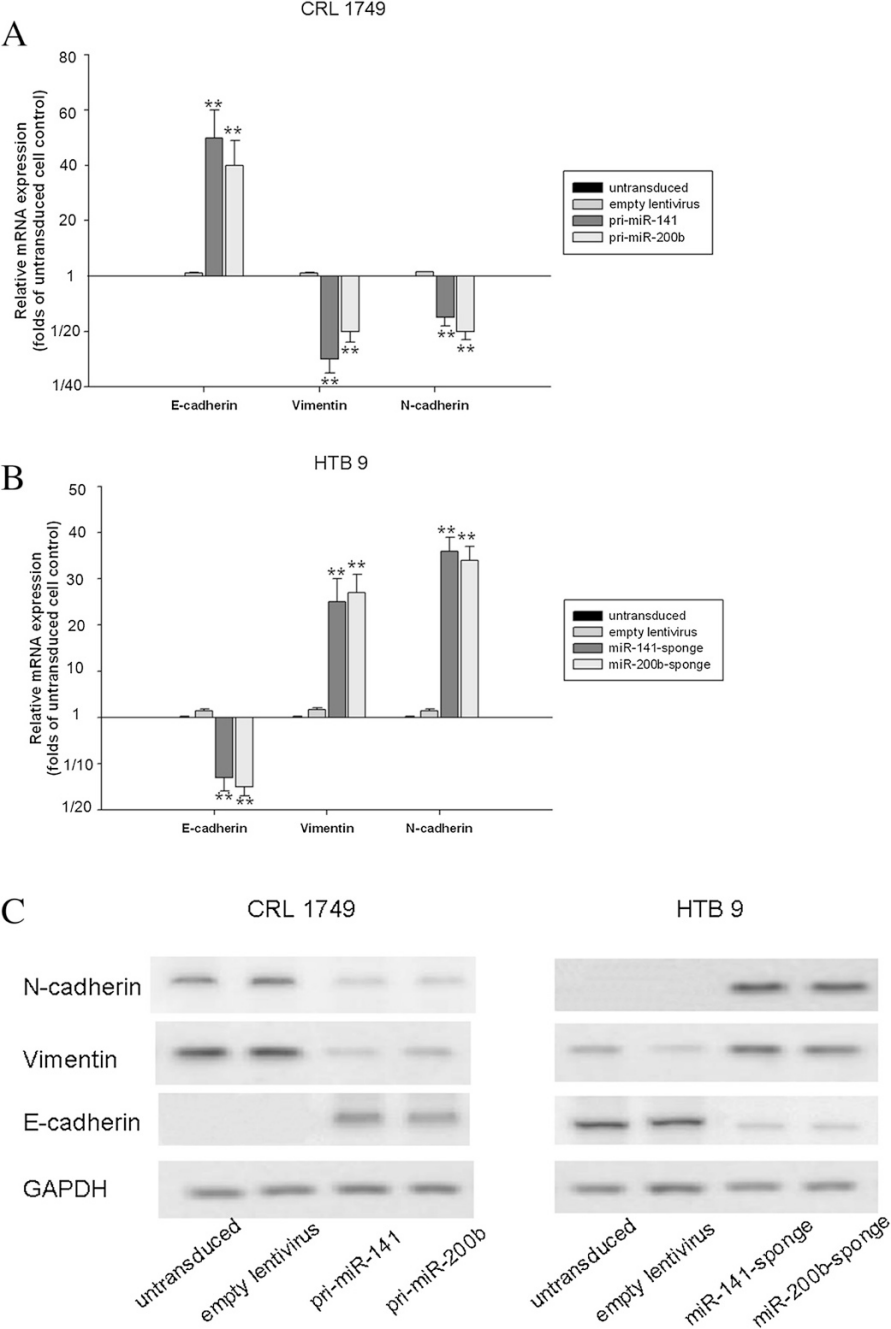
**After the stable lentiviral transduced cell lines were established, CRL 1749 and HTB9 cells were subjected to RT-PCR and western blotting.** The markers for EMT included E-cadherin, vimentin and N-cadherin shown in the **A** (CRL 1749 cells) and **B** (HTB9 cells) showed the pooled data of (mean ± S.E.M) from three independent experiments for mRNA expression. **C** demonstrated the results of western blotting analysis from two cell lines.

The expression of the epithelial marker E-cadherin at mRNA and protein levels increased significantly in CRL 1749 cells following overexpression of miRNA-141 and miRNA-200b, whereas the expression of mesenchymal markers, including vimentin and N-cadherin, was downregulated (Figure 3A and C). In contrast, E-cadherin expression at both mRNA and protein levels declined sharply in HTB 9 cells when expression of miRNA-141 and miRNA-200b was inhibited using the sponges of both miRNAs (Figure 3B and C). Further, as we expected, trends with both vimentin and N-cadherin were opposite to that of E-cadherin: their expression was consistently elevated at mRNA and protein levels, as shown in Figure 3B and C, when miRNA-141 and miRNA-200b were inhibited.

### The predictive role of miRNA-141 and miRNA-200b in PLND

A total of 78 patients with BC who underwent cystectomy and super-extended PLND were included in this study. Their clinical characteristics are shown in Table 1. According to radiological tests and classification by CTx stage, 60 were defined as cT1-2 and 18 as cT3-4. On histopathological examination, 51 patients were diagnosed with pT1-2 bladder carcinoma and 27 patients with pT3-4 bladder carcinoma. The pathological examination of corresponding harvested lymph nodes was performed separately. Positive lymph nodes were found in 23 patients, among whom 16 had positive nodes limited to the level I region, 6 had lymph node metastasis at level I and II, and 1 had level I+ II+ III node metastasis.

**Table 1:** **The clinical characteristics of BC patients received cystectomy and super-extended PLND**

No. patients	78
Mean (SD) age, years	57 (12.7)
No (%) of males	64 (82.1)
cT	
≤pT2b	60
≥pT3a	18
pT	
≤pT2b	51
≥pT3a	27
Mean (range) lymph nodes retrieved	25.7 (8–39)
LN+	23 (29.5%)
Level I	16 (20.5%)
Level I + II	6 (7.7%)
Level I + II + III	1 (1.3%)

SD: Standard deviation.

**Figure 4:**
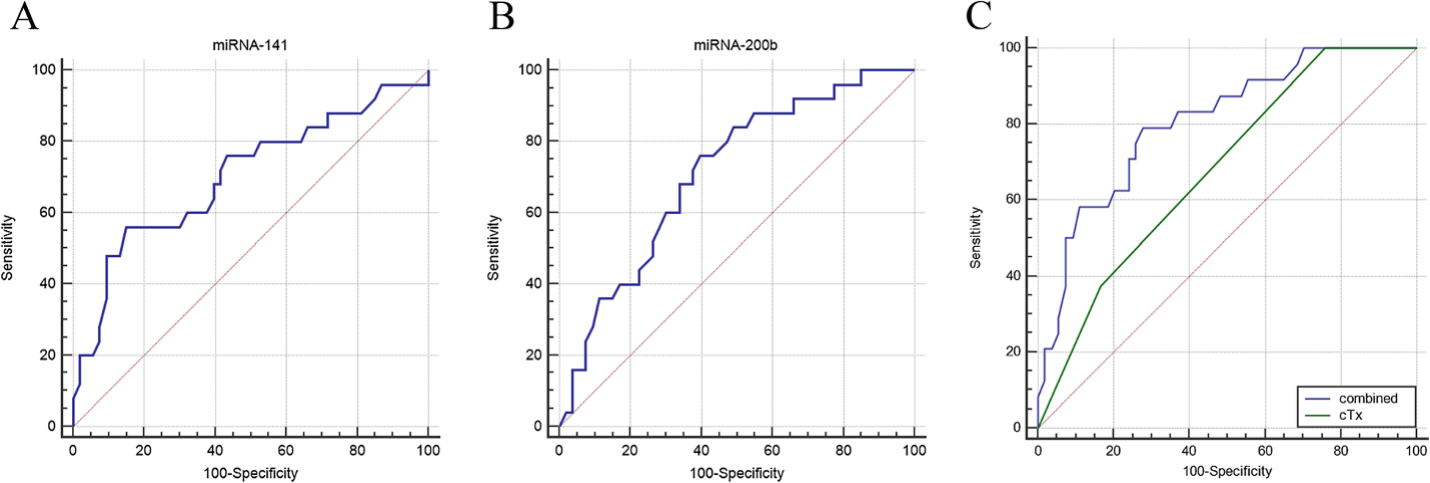
**ROC curves used to evaluate the diagnostic potential of (A) miRNA-141(B) miRNA-200b (C) combined model (cTx, miRNA-141 and miRNA-200b).**

Seventy-eight urine samples were collected before operation and analyzed for the expression of miRNA-141 and miRNA-200b. The patients were classified according to the presence of lymph node metastasis (54 lymph node negative, 23 lymph node metastasis positive). To evaluate the diagnostic potential, ROC curves were generated for miRNA-141 and miRNA-200b (Figure 4A and B, respectively). The ROC curves show that both miRNA-141 (AUC = 0.704, 78.2% sensitivity, 51.6% specificity) and miRNA-200b (AUC = 0.674, 81.3% sensitivity, 47% specificity) discriminated between patients who had lymph node metastasis and those who were lymph node negative, confirming the predictive role of urine miRNA-141 and miRNA-200b. According to the optimal cut-off on the ROC curve, the levels of expression of miRNA-141 and miRNA-200b were categorized into low and high expression. The relationships between miRNA expression and clinical characteristics are shown in Additional file 2: Table S2. Tumors with low levels of expression of miRNA-141 are more invasive (pT3-4). Expression of miRNA-141 and miRNA-200b was found to be associated with lymph node metastasis.

In previous studies, we concluded that CT staging can be used as a decision-making marker for the extent of lymph node dissection [19]. According to the results of the earlier studies, the probability of lymph node metastasis at stages cT1-2a, cT2b and cT3-4 is sequentially increased. Therefore, we divided the patients into several groups on the basis of CT staging and miRNA expression. As shown in Table 2, low-risk patients (cT1-2a, cT2b with high miRNA-141 and miRNA-200b expression) rarely had positive lymph nodes (1/22), while the remaining high-risk patients (cT2b with low miRNA-141 and/or miRNA-200b expression, cT3-4) were prone to having LN+ (22/56). A ROC curve was generated to compare the predictive value of CT staging and the combined model (cTx, miRNA-141 and miRNA-200b). The ROC curves showed that the combined model (AUC = 0.749, 95% CI = 0.668–0.882) can discriminate better between patients with lymph node metastasis and those without than CT staging alone (AUC = 0.679, 95% CI = 0.564–0.781) (P < 0.05, Figure 4C).

**Table 2:** **Classification of bladder cancer patients based on CT staging and miRNA expression**

Group	Content	n	LN+	Level I	Level I + II	Level I + II + III
1	cT1-2a	12	0	0	0	0
2	cT2b + high*	10	1	1	0	0
3	cT2b + low**	31	14	10	4	0
4	cT3-4 + high	4	2	1	1	0
5	cT3-4 + low	13	6	4	1	1
1-2	Low risk	22	1	1	0	0
3-5	High risk	56	22	15	6	1

*High represents high expression of miRNA-141 and miRNA-200b.

**Low represents low expression of miRNA-141 and/or miRNA-200b.

## Discussion

Recently, increasing numbers of studies have revealed a direct relationship between the miRNA-200 family and bladder cancer [5,11-13]. In this study, we first tried to explore the underlying mechanisms using in vitro experiments.

The invasive ability of cancer cells changed greatly after manipulation of expression of miRNA-141 and miRNA-200b. The invasiveness and migration of CRL 1749 cells were inhibited by increased expression of miRNA-141 or miRNA-200b, while HTB9 cells showed the opposite response after downregulation of miRNA-141 or miRNA-200b. The MMPs promote cancer progression by boosting cancer cell growth and migration, with invasion and metastasis. However, few researchers have studied the relationship between miRNA and the expression of MMPs in bladder cancer. Liu et al. [22] found that MMP-2 and MMP-9 were significantly downregulated in HTB9 cells overexpressing miRNA-430. In our study, the activity of the MMP-2 and MMP-9 enzymes was reduced markedly when the expression of miRNA-141 or miRNA-200b was increased in CRL 1749 cells. In contrast, enzyme activity was upregulated in HTB9 cells after expression of miRNA-141 or miRNA-200b decreased. We have previously confirmed that MMP-16 is a novel direct downstream functional target of miRNA-200b in two bladder cell lines. Not only can MMP-16 degrade some matrix molecules directly, but it also activates other MMPs (MMP-2 and-9) [23,24]. The results mentioned here have been submitted to an academic journal for possible publication.

It has been shown that the miRNA-200 family is a powerful regulator of EMT [25]. Consistent with their role in the regulation of EMT, changes in miRNA-141 and miRNA-200b expression could lead to corresponding changes in EMT phenotype. The data of Kenney et al. [26] show that expression of vimentin and N-cadherin was reduced after they forcibly increased the intracellular level of miRNA-141 in CRL 1749 cells. In our study, the expression of E-cadherin increased significantly in CRL 1749 cells after miRNA-141 and miRNA-200b were overexpressed, whereas the expression of vimentin and N-cadherin, the mesenchymal markers, was downregulated. In contrast to the overexpression of miRNA-141 and miRNA-200b in CRL 1749 cells, these EMT marker proteins showed the opposite trends in HTB9 cells when the expression of miRNA-141 and miRNA-200b was downregulated.

These results reveal that modulation of expression of miRNA-141 and miRNA-200b leads to changes in invasive ability, which is mechanistically associated with the EMT phenotype of two bladder cancer cell lines. We further investigated their potential as biomarkers for deciding the extent of PLND necessary during cystectomy.

Four templates of PLND have been well defined: limited, standard, extended and super-extended. Recently, super-extended PLND has been recommended by many urologists for all patients undergoing cystectomy [8,9]. Super-extended PLND is considered to be associated with better disease-free survival for BC patients with endopelvic lymph node involvement [27]. The rate of lymph node metastasis increases from 5% in non-muscle-invasive bladder tumors (pT0, pTa, pTis, pT1) to 18% in superficial muscle-invasive tumors (pT2a), 27% in deep muscle-invasive tumors, and 45% in extravesical tumors (pT3-4). Therefore, many urologists have used clinical staging to predict lymph node metastasis [19,28]. Shariat et al. [28] developed a model that uses the clinical (preoperative) tumor stage to determine the number of nodes needed to be removed at radical cystectomy to determine the true nodal status. Our previous study also demonstrated the predictive role of preoperative tumor staging by CT in patients with lymph node metastasis [19].

In this study, we explored the relationship between urinary miRNA-200 expression and lymph node metastasis in patients with BC for the first time, and found that urine miRNA expression can predict lymph node metastasis. This predictive or prognostic effect of the miRNA-200 family has also been described for other tumors [29,30]. Circulating levels of miRNA-200b were found to be an independent predictor of overall survival in patients with prostate cancer who received docetaxel chemotherapy [29]. Toiyama et al. reported that serum miRNA-200c has strong potential to serve as a noninvasive biomarker for colorectal cancer prognosis and prediction of metastasis [30]. In addition, loss of expression of the miRNA-200 family is associated with poor prognosis in patients with gastric or breast cancer [31-33]. Given the heterogeneity of BC, it is improbable that a single marker or tumor stage alone can accurately segregate tumors into distinct aggressive categories. A combination of CT stage with miRNA-141 and miRNA-200b levels was a more appropriate model to predict lymph node metastasis than use of each marker alone (Figure 2). This predictive model achieved 88% sensitivity and 43.4% specificity, suggesting that the miRNA-141 and miRNA-200b could be promising biomarkers for deciding the extent of PLND necessary during cystectomy.

The present study was not without limitations. First, levels of miRNA-141 and miRNA-200b may not be the only appropriate urine biomarkers for prediction of LN+. Our decision to use these markers was based on the key role of the miRNA-200 family in EMT. In the future, other confirmed predictive urine biomarkers can be added to miRNA-141 and miRNA-200b, which may improve the predictive accuracy of the combined model. Second, some potential confounding factors, such as smoking, may affect the accuracy of the AUC curve, but the impact cannot be evaluated precisely. Third, the sample size of present study was relatively small and was not sufficient to provide definite conclusions for decision-making regarding biomarkers for PLND, especially with the use of subgroup analysis.

## Conclusion

MiRNA-141 and miRNA-200b play important roles in the invasive ability and EMT phenotype of bladder cancer. Detection of miRNA-141 and miRNA-200b can help to identify patients undergoing cystectomy who are likely to have lymph node metastasis and would therefore potentially benefit from super-extended PLND.

## Additional files

Additional file 1: Table S1. miR-200 deregulation in bladder cancer tissues and adjacent tissues.
Additional file 2: Table S2. Relationship between miRNA expression and clinical characteristic.
