# Supplementary material for: MiRNA-141 and miRNA-200b are closely related to invasive ability and considered as decision-making biomarkers for the extent of PLND during cystectomy
Source: BMC Cancer. 2015 Mar 4;15:92. doi: 10.1186/s12885-015-1110-7 (PMC4350852; doi:10.1186/s12885-015-1110-7)
Supplement: Additional file 1: Table S1. — miR-200 deregulation in bladder cancer tissues and adjacent tissues. [file 12885_2015_1110_MOESM1_ESM.doc]

Table S1 miR-200 deregulation in bladder cancer tissues and adjacent tissues.

| miRNA | Fold change(tumor/adjacent) | p value |
| --- | --- | --- |
| hsa-miR-200a | -2.14 | <0.05 |
| hsa-miR-200b* | -4.28 | <0.01 |
| hsa-miR-200c | -1.86 | <0.05 |
| hsa-miR-141* | -3.25 | <0.01 |
| hsa-miR-429 | -1.41 | >0.05 |

* selected miRNA for further study.
