# Supplementary material for: MiRNA-141 and miRNA-200b are closely related to invasive ability and considered as decision-making biomarkers for the extent of PLND during cystectomy
Source: BMC Cancer. 2015 Mar 4;15:92. doi: 10.1186/s12885-015-1110-7 (PMC4350852; doi:10.1186/s12885-015-1110-7)
Supplement: Additional file 2: Table S2. — Relationship between miRNA expression and clinical characteristic. [file 12885_2015_1110_MOESM2_ESM.doc]

Table S2 Relationship between miRNA expression and clinical characteristic.

| Clinical | n | miR-141 | | p |  | miR-200b | | p |
| --- | --- | --- | --- | --- | --- | --- | --- | --- |
| features | 78 | High  (n=29) | Low  (n=49) |  |  | High  (n=30) | Low  (n=48) |  |
| Sex |  |  |  | >0.05 |  |  |  | >0.05 |
| Male | 64 | 24 | 40 |  |  | 23 | 41 |  |
| Female | 14 | 5 | 9 |  |  | 7 | 7 |  |
| Age(years) |  |  |  | >0.05 |  |  |  | >0.05 |
| <60 | 38 | 13 | 25 |  |  | 12 | 26 |  |
| >60 | 40 | 16 | 24 |  |  | 18 | 22 |  |
| cT |  |  |  | >0.05 |  |  |  | >0.05 |
| ≤pT2b | 60 | 23 | 37 |  |  | 22 | 38 |  |
| ≥pT3a | 18 | 6 | 12 |  |  | 8 | 10 |  |
| pT |  |  |  | <0.05 |  |  |  | >0.05 |
| ≤pT2b | 51 | 26 | 27 |  |  | 21 | 30 |  |
| ≥pT3a | 27 | 5 | 22 |  |  | 9 | 18 |  |
| pN |  |  |  | <0.05 |  |  |  | <0.05 |
| N0 | 55 | 26 | 29 |  |  | 26 | 29 |  |
| LN+ | 23 | 3 | 20 |  |  | 4 | 19 |  |
| LN I | 16 | 2 | 14 |  |  | 3 | 13 |  |
| LN II | 6 | 1 | 5 | >0.05* |  | 2 | 4 | >0.05* |
| LN III | 1 | 0 | 1 | >0.05* |  | 0 | 1 | >0.05* |
| miR-141 |  |  |  |  |  |  |  | >0.05 |
| High | 29 |  |  |  |  | 10 | 19 |  |
| Low | 49 |  |  |  |  | 20 | 29 |  |
| miR-200b |  |  |  | >0.05 |  |  |  |  |
| High | 30 | 9 | 21 |  |  |  |  |  |
| Low | 48 | 20 | 28 |  |  |  |  |  |

* compared with LN I.
